# Redefining ARDS phenotypes: Challenges in the precision medicine era

**DOI:** 10.1016/j.jointm.2025.08.002

**Published:** 2025-09-10

**Authors:** Raffaele Merola, Denise Battaglini

**Affiliations:** 1 Anesthesia and Intensive Care Medicine, Department of Neurosciences, Reproductive and Odontostomatological Sciences, University of Naples Federico II, Naples, Italy; 2 Department of Surgical Sciences and Integrated Diagnostics (DISC), University of Genoa, Genova 16132, Italy; 3 Anesthesia and Intensive Care, IRCCS Ospedale Policlinico San Martino, Genova 16132, Italy

In recent years, the concept of phenotyping in acute respiratory distress syndrome (ARDS) – where ARDS is viewed as a broad phenotype encompassing multiple sub-phenotypes – has gained considerable interest.^[^^1^^]^ Machine learning and statistical clustering analyses of data from five randomized controlled trials (RCTs) have identified distinct ARDS subtypes –primarily hyperinflammatory and hypoinflammatory profiles –based on clinical characteristics and blood biomarkers associated with coagulopathy, endothelial injury, and inflammation. These subtypes not only exhibit differences in clinical outcomes and may respond variably to therapeutic interventions.^[^[2], [3], [4], [5], [6]^]^ Characterized by elevated plasma levels of pro-inflammatory cytokines (including tumor necrosis factor [TNF-α], interleukin [IL]-6, and IL-8), increased markers of endothelial damage, more severe shock, and a higher prevalence of metabolic acidosis, the hyperinflammatory sub-phenotype exhibits a significantly higher mortality rate compared to hypoinflammatory phenotype. Crucially, these phenotypes exhibit differential responses to ventilatory strategies, fluid management, and pharmacological interventions. The hyperinflammatory phenotype appears to benefit more from higher levels of positive end-expiratory pressure (PEEP) and conservative fluid management strategies. Similarly, this phenotype appears more likely to benefit from immunomodulatory therapies, including corticosteroids, potentially due to their heightened inflammatory milieu. In contrast, patients with the hypoinflammatory phenotype may not respond favorably to such interventions and could even be harmed by them.^[^[2], [3], [4], [5], [6]^]^

These developments have fostered optimism about moving toward precision medicine in ARDS, a syndrome long recognized for its heterogeneity and poor response to therapies.^[^^7^^,^^8^^]^

Nevertheless, a closer examination of the terminology used reveals significant conceptual ambiguities that may hinder, rather than advance, clinical translation. In biology, a phenotype refers to the set of observable traits of an organism resulting from the interaction between its genotype and environmental factors. This idea frequently assumes that genetic variation and phenotypic expression are linked mechanistically through specific biological pathways. This approach is still widely used in disciplines like cancer and Mendelian disorders: some genetic abnormalities result in predictable treatment vulnerabilities and repeatable clinical presentations. Phenotypes in these situations are not only descriptive; they are also defined mechanistically.

By contrast, in ARDS, the observable trait – acute hypoxemic respiratory failure (AHRF) – is nearly identical across patients, despite diverse precipitating insults. Whether the precipitant is pneumonia, sepsis, pancreatitis, or trauma, the clinical syndrome converges toward the same clinical manifestation.^[^^9^^]^ What varies is the interaction that gives rise to the syndrome – namely, the underlying cause and the host’s individual biological response. However, our current understanding of the genomic or transcriptomic determinants of these interactions remains limited.

To capture this biological heterogeneity, the concept of endotype – a subtype of a condition defined by a distinct pathobiological mechanism – offers a more appropriate framework for understanding heterogeneity in ARDS.^[^^10^^]^ Several distinct pathways may lead to the same clinical phenotype. For example, direct (e.g., pneumonia, aspiration) and indirect (e.g., non-pulmonary sepsis, trauma) forms of lung injury may both result in diffuse alveolar damage, but through differing pathways of endothelial injury, immune activation, and cellular signaling. Within these endotypes, biological subtypes may further emerge based on immune or inflammatory profiles, often identified via machine learning or clustering techniques.^[^^11^^,^^12^^]^ These may be better termed biotypes, as they reflect differing biological states rather than observable phenotypes.

Endotypes, however, only reflect one stratification level. A variety of genetic and environmental factors combine to form the clinically evident illness known as the wider ARDS phenotype. Based on repeatable clinical or biological traits that distinguish one patient group from another across several domains, sub-phenotypes are discovered within the ARDS phenotype. Subgroups, on the other hand, are frequently defined by single-variable thresholds (e.g., driving pressure, PaO₂/FiO₂ ratio) and may be more arbitrary or context-dependent. A hierarchical model for deciphering ARDS heterogeneity and directing customized treatment approaches is offered by these stratification levels taken together, which range from phenotype to sub-group to sub-phenotype and endotype.

Therefore, naming these subtypes as “sub-phenotypes” may be premature and potentially misleading. Without clear causal links between genotype, pathobiology, and clinical expression, we risk misclassifying patterns of correlation as distinct disease types.

These stratification levels – phenotype to sub-group, sub-phenotype, and endotype – combine to provide a hierarchical model that attempts to explain the variability of ARDS and direct individualized treatment plans. However, this paradigm is further complicated by the syndrome’s temporal dynamics. Since ARDS is a syndrome that is always changing, its symptoms and even its designation as a subtype may also change over time.^[^^13^^]^ The designation of specific patient clusters as “sub-phenotypes” may therefore be premature and, in certain situations, deceptive. Transient or correlative patterns may be misinterpreted as distinct disease entities when causal linkages between genetic profiles, underlying pathobiology, and “actual” clinical manifestations remain unestablished.

Terminological ambiguity has tangible consequences: It shapes trial methodology, influences the interpretation of findings, and impacts the selection of therapeutic targets. Moreover, the excitement surrounding “phenotyping” may obscure the fact that most subgroup definitions are based on retrospective correlation rather than mechanistic understanding and are not yet actionable at the bedside.^[^^14^^]^ Despite its clinical significance, the hyperinflammatory subtype – which shows higher mortality and may exhibit different responses to therapies such as fluid restriction or statins – remains difficult to detect at the bedside and lacks a standardized biomarker definition.^[^^3^^,^^6^^]^

In an effort to align mechanistic precision with bedside applicability, we advocate for a stepwise stratification of ARDS encompassing phenotypes, endotypes, and biotypes. This model (Figure 1) aligns with established practices in diseases like asthma and provides a structured pathway for biomarker development, therapeutic targeting, and trial enrichment.^[^^15^^]^

**Figure 1:**
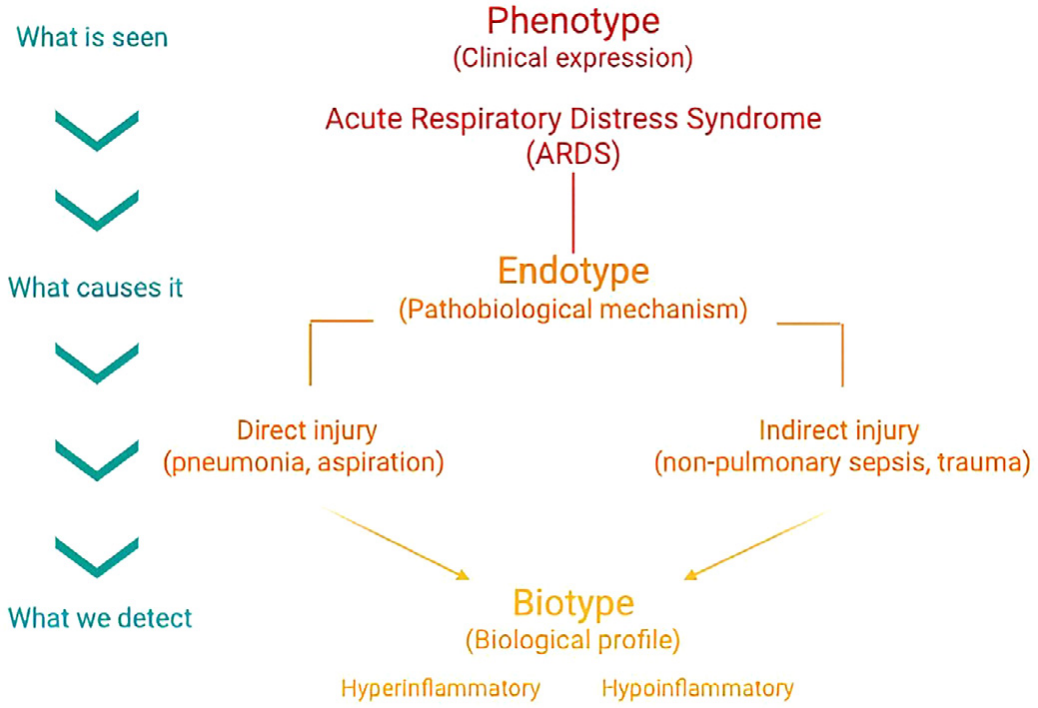
Conceptual hierarchy for ARDS stratification. ARDS: Acute respiratory distress syndrome.

## Take-home Message

The pursuit of precision medicine to individualize care in ARDS is both necessary and promising. However, precision begins with both conceptual and biological clarity. As we continue to unravel the heterogeneity of this syndrome, adopting accurate terminology is not an academic exercise, but a prerequisite for progress. The use of the term “phenotyping” often suggests an underlying mechanistic clarity that remains largely elusive in the context of ARDS. Pursuing efforts to subtype ARDS itself is not worthwhile, given that many different diseases can cause it; instead, the focus should be on subtyping the underlying diseases that lead to its development. Rather than creating new ARDS subtypes, we should base our research on established categories – endotypes (direct *vs.* indirect lung injury) and biotypes (hyperinflammatory *vs.* hypoinflammatory) – and build the science around these frameworks. Conceptual clarity is not a barrier to innovation; it is its foundation.

## CRediT authorship contribution statement

**Raffaele Merola:** Writing – review & editing, Writing – original draft, Visualization, Validation, Project administration, Methodology, Investigation, Conceptualization. **Denise Battaglini:** Writing – review & editing, Writing – original draft, Visualization, Validation, Supervision, Project administration, Methodology, Investigation.
